# Vaccination Against Influenza and Pneumococcus During Pretravel Health Consultations in the United States: Interventions and Missed Opportunities

**DOI:** 10.1093/ofid/ofae761

**Published:** 2025-01-07

**Authors:** Loukas Kakoullis, Sowmya R Rao, Edward T Ryan, Allison T Walker, Lin H Chen, Regina C LaRocque

**Affiliations:** Department of Medicine, Mount Auburn Hospital, Cambridge, Massachusetts, USA; Harvard Medical School, Boston, Massachusetts, USA; Department of Global Health, Boston University School of Public Health, Boston, Massachusetts, USA; Harvard Medical School, Boston, Massachusetts, USA; Travelers’ Advice and Immunization Center, Massachusetts General Hospital, Boston, Massachusetts, USA; Division of Infectious Diseases, Massachusetts General Hospital, Boston, Massachusetts, USA; Department of Immunology and Infectious Diseases, Harvard T. H. Chan School of Public Health, Boston, Massachusetts, USA; Travelers’ Health Branch, Division of Global Migration Health, Centers for Disease Control and Prevention, Atlanta, Georgia, USA; Department of Medicine, Mount Auburn Hospital, Cambridge, Massachusetts, USA; Harvard Medical School, Boston, Massachusetts, USA; Harvard Medical School, Boston, Massachusetts, USA; Travelers’ Advice and Immunization Center, Massachusetts General Hospital, Boston, Massachusetts, USA; Division of Infectious Diseases, Massachusetts General Hospital, Boston, Massachusetts, USA

**Keywords:** *Streptococcus pneumoniae*, flu vaccine, GTEN, travel medicine

## Abstract

**Background:**

Infections by *Streptococcus pneumoniae* and influenza viruses are vaccine-preventable diseases causing great morbidity and mortality. We evaluated pneumococcal and influenza vaccination practices during pre–international travel health consultations.

**Methods:**

We evaluated data on pretravel visits over a 10-year period (1 July 2012 through 31 June 2022) from 31 sites in Global TravEpiNet (GTEN), a consortium of US healthcare facilities providing pretravel health consultations. Data were collected using an online structured questionnaire utilized by GTEN providers. We obtained summary statistics and performed multivariable logistic regression models to identify characteristics associated with receiving the vaccinations.

**Results:**

At 116 865 pretravel visits, 28 754 (25%) travelers were eligible to receive pneumococcal vaccination and 56 150 (48%) travelers were eligible to receive influenza vaccination. A total of 19 557 (68%) pneumococcal vaccine–eligible travelers were not offered the vaccine at the pretravel visit. Among influenza vaccine–eligible travelers, 8592 (15%) were not offered the vaccine, and an additional 16 931 (30%) travelers declined the vaccine. Influenza vaccine was not available for 8014 (14%) eligible travelers. Nonadministration of the influenza vaccine was most frequent in the months of April through September. Compared to nonacademic centers or centers in the South or Midwest, travelers seen in academic centers or centers in the Northeast were more likely to receive either vaccine.

**Conclusions:**

Increasing awareness of global influenza transmission patterns and improving access to routine vaccines at the pretravel encounter may enhance vaccination for respiratory pathogens in departing US international travelers.

## Teaser

Analyses of pretravel health consultations at 31 US sites over 10 years reveals opportunities to increase pneumococcal and influenza vaccinations. Increasing awareness of global influenza transmission patterns and improving access to routine vaccines at pretravel encounters may enhance vaccination rates.

Infections of the respiratory tract are among the leading causes of mortality worldwide. In 2019, *Streptococcus pneumoniae* and influenza viruses were the 2 leading vaccine-preventable respiratory causes of death globally in adults, estimated to have claimed more than 1 380 000 and 240 000 lives, respectively [1]. Influenza is a top travel-related risk, with an estimated incidence of 1% per month of travel [2]. Another important respiratory tract infection in travelers is severe acute respiratory syndrome coronavirus 2 (SARS-CoV-2), which was the third leading cause of death in the United States (US) in 2020 and 2021 [3], and the fourth leading cause of death in 2022 [4].

Vaccinations are effective in limiting the impact of respiratory diseases. Pneumococcal vaccination prevents invasive pneumococcal disease as well as pneumococcal pneumonia [5, 6]. Influenza vaccination prevents influenza disease and influenza-associated hospitalizations, intensive care unit admissions, and mortality [7]. Formulations for influenza vaccine are updated annually to optimize effectiveness against changes in circulating strains variants [8]. New pneumococcal vaccines have been introduced to broaden effectiveness against more pneumococcal serotypes [9].

A pretravel health encounter focuses on travel-related counseling and vaccination and also presents an opportunity to ensure that travelers are up-to-date on routine vaccinations. Given the public health importance of pneumococcal and influenza vaccination, we sought to evaluate the clinical practice of travel medicine providers with regard to providing these 2 vaccinations in Global TravEpiNet (GTEN), a US-based consortium of pretravel health providers [10].

## METHODS

### GTEN Data

We evaluated data from pretravel health consultations conducted at GTEN sites [10]. During the pretravel consultation, travelers and providers in GTEN used an online structured questionnaire, as previously described [10]. Travelers entered information into the structured questionnaire regarding their age, medical conditions, and travel itinerary (eg, destination[s], reason for travel, and duration of travel). Providers confirmed the information entered by the traveler and then entered data about immunization history, health advice provided, vaccines administered, and medications prescribed during the pretravel consultation. Travelers specified their reasons for travel, which we categorized into 6 categories in the following rank order: visiting friends and relatives (VFR), business, humanitarian service (including providing medical care, nonmedical service work, and missionary work), research/education, leisure, and other. Travelers who selected leisure along with a second purpose of travel (eg, VFR) were included only in the second category. For pediatric travelers, parents or guardians provided the self-reported information, including the reason for family travel, which could include any of the reasons above.

At each visit, providers assessed the travelers' immunization history in accordance with their routine clinical practice. For influenza, the structured GTEN questionnaire required providers to consider vaccination for all travelers who had not already received the seasonal influenza vaccine or for whom the vaccine was not ordered. For pneumococcal vaccination, the structured GTEN questionnaire required providers to consider vaccination for all travelers if they had not previously received pneumococcal vaccination in accordance with the relevant Advisory Committee on Immunization Practices (ACIP) recommendations at the time of their visit, or for whom the vaccine was not ordered by the provider at the pretravel visit. The structured GTEN questionnaire prompted providers to select 1 of the following reasons for not vaccinating the traveler: (1) referral to another provider for vaccination, (2) vaccination not indicated for the itinerary, (3) preexisting immunity, (4) traveler refusal, (5) medical contraindication, (6) vaccine unavailable at the clinic, or (7) insufficient time for vaccination before departure. Incomplete answers were not permitted [11, 12]. Answer options were mutually exclusive, permitting the provider to select only a single answer.

We included data on all international travelers evaluated at any of the 31 GTEN sites from 1 July 2012 through 30 June 2022. Institutional review boards at each of the participating GTEN sites either approved the study or considered it exempt from review.

### Vaccine Eligibility

For this analysis, we considered travelers to be “influenza vaccine eligible” if they were 6 months of age or older and had not received a current influenza seasonal vaccine. Since influenza seasonality varies geographically, we characterized influenza vaccine eligibility in a secondary analysis by the influenza seasonality at the traveler’s destination, using data published by the World Health Organization and other sources [13–17]. Destinations were categorized into 4 groups based on their influenza seasonality as follows: April–September, October–March, year-round, and unclassified [17]. Travel dates and destinations were analyzed to evaluate whether the travel was taking place during influenza season at the destination. We further focused on visits that took place in July and August, under the assumption that the influenza vaccine is not available in the US during these months.

We considered travelers to be “pneumococcal vaccine eligible” if they were <5 years of age or ≥65 years of age or had an underlying condition for which ACIP recommendations specified an indication for pneumococcal vaccination including a history of smoking, chronic cardiovascular disease, chronic lung disease, chronic liver disease, or having an immunocompromising condition [18], and they had not been vaccinated in the past. Due to the variety of available pneumococcal vaccines over the study period, we considered administration of any type of pneumococcal vaccine to be appropriate vaccination for the purposes of this analysis.

### Statistical Analysis

We obtained summary statistics on traveler characteristics and use of vaccine among those considered eligible. We categorized travelers who were referred to another provider for vaccination or vaccinated during the visit as “vaccinated” and those for whom the vaccine was not available at the clinic, the traveler declined, or the provider considered the vaccine as not indicated for the itinerary as “not vaccinated.” We calculated odds ratios (ORs) and 95% confidence intervals (CIs) from separate multivariable logistic regression models to assess the relationship of traveler characteristics with “not being vaccinated” for the given vaccine. Only patients who were considered eligible for each vaccine were entered into each corresponding model. Models included gender; age in years; presence of 1 or more medical conditions; travel to Africa, Southeast Asia, South America, Western Pacific, Central America/Caribbean, Eastern Mediterranean, or Europe; VFR travel; duration of travel in days; US census region of the GTEN; site; and type of site. The bivariate distributions (numbers and percentages) of the characteristics by vaccine administration status and ORs with 95% CIs obtained from the multivariable regressions are presented as forest plots. All analyses were conducted in SAS version 9.4 software (SAS Institute, Cary, North Carolina) and a 2-sided *P* < .05 was considered significant.

## RESULTS

### Traveler Demographics and Travel Characteristics

A total of 116 865 pretravel health consultations took place at 31 GTEN sites during the 10-year study period. Of the travelers evaluated, 66 293 (57%) were female. The mean age of travelers was 38 years, and the most common region visited was Africa (42%) (Table 1). Most travelers (68 737 [59%]) were traveling for 2 weeks or longer.

**Table 1.** Demographic and Travel Characteristics of Travelers Seen at Global TravEpiNet Sites, 2012–2022 (N = 116 865)

Characteristic	No.^a^ (%)
Gender	
Female	66 293 (57)
Male	50 572 (43)
Age category	
<65 y	104 474 (89)
≥65 y	12 391 (11)
Past medical history^b^	
Cardiovascular disease	21 020 (18)
Endocrine disease	10 353 (9)
Lung disease	9474 (8)
Cancer or blood disorder	7328 (6)
Immune system disease	4640 (4)
Kidney disease	2087 (2)
Liver disease	1495 (1)
≥1 medical condition	34 365 (29)
≥2 medical conditions	38 026 (33)
No medical conditions	44 474 (38)
WHO region^b^	
Africa	48 565 (42)
Southeast Asia	24 239 (20)
South America	19 061 (16)
Western Pacific	18 062 (16)
Central America/Caribbean	14 561 (13)
Eastern Mediterranean	6231 (5)
Europe	5725 (5)
Reason for travel^b^	
Leisure only	50 620 (43)
Business	16 974 (15)
Returning to region of origin of self or family to visit friends and relatives	11 862 (10)
Research/education	11 068 (10)
Nonmedical service work	7985 (7)
Missionary service	7352 (6)
Adventure (eg, mountaineering, water sports, outdoor activities)	7169 (6)
Providing medical care	4694 (4)
Type of destination	
Urban	34 309 (29)
Rural	12 799 (11)
Both	69 757 (60)
Type of accommodation^b^	
Hotel	79 687 (68)
Home stay with relatives	20 518 (18)
Dormitory or hostel	17 589 (15)
Other	16 647 (14)
Home stay with nonrelatives	13 620 (12)
Camping (not including luxury safari accommodations)	8049 (7)
Cruise	5050 (4)
Duration of travel	
≥14 d	68 737 (59)
1–13 d	48 077 (41)
US census regional classification of GTEN site	
Northeast	54 565 (47)
South	31 192 (27)
West	26 663 (23)
Midwest	4445 (4)
Type of GTEN site	
Nonacademic center	17 126 (15)
Academic center	99 739 (85)

Data are presented as No. (%).

Abbreviations: GTEN, Global TravEpiNet; US, United States; WHO, World Health Organization.

^a^May not add to 100% due to missing observations.

^b^Not mutually exclusive categories.

### Clinical Approach to Pneumococcal Vaccine–Eligible Travelers

Out of 116 865 travelers receiving pretravel health consultations, 28 754 (25%) were eligible to receive pneumococcal vaccine, based on their past medical history and smoking status (Table 2). Of the pneumococcal vaccine–eligible travelers, 5320 (19%) were vaccinated at the pretravel health encounter or referred to another provider for vaccination. However, 21 434 (74%) of the pneumococcal vaccine–eligible travelers were not vaccinated at the pretravel health encounter or referred; in most instances (19 557 [68%]), travelers were not vaccinated because the provider selected that the vaccine was not indicated for the itinerary; an additional 1838 (6%) travelers were offered the vaccine but declined.

**Table 2.** Clinical Approach to Influenza Vaccine–Eligible and Pneumococcal Vaccine–Eligible Travelers Evaluated at Global TravEpiNet Sites, 2012–2022

Vaccination Status	Pneumococcal Vaccine Eligible (n = 28 754)	Influenza Vaccine Eligible (n = 56 150)
Vaccinated or referred	5320 (19)	22 613 (40)
Referred to another provider for administration	3588 (12)	6955 (12)
Vaccinated during the visit	1732 (6)	15 658 (28)
Not vaccinated^a^	21 434 (74)	33 537 (60)
Not indicated for the itinerary	19 557 (68)	8592 (15)
Traveler declined	1838 (6)	16 931 (30)
Vaccine not available	39 (<1)	8014 (14)

Data are presented as No. (%).

^a^Categories with <1000 travelers for both vaccines, which included “insufficient time to complete prior to departure” and “medical contraindication,” are omitted.

**Figure 1.**
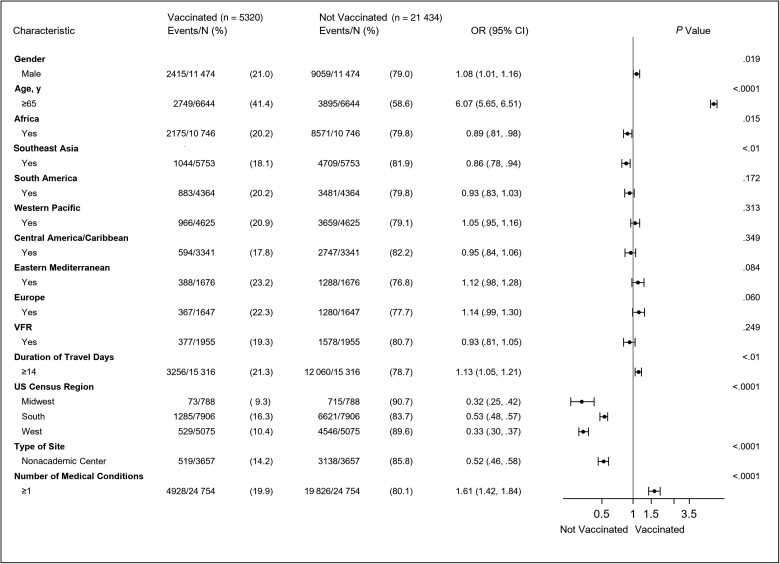
Forest plot depicting the results obtained from a multivariable logistic regression evaluating the relationship between traveler characteristics and receipt of the pneumococcal vaccine among persons eligible to receive the vaccine at the pretravel health encounter. Model included all characteristics shown in the figure. Reference categories: Gender: female; Age in years: <65; travel to each geographic region: no; Visiting friends and relatives: no; duration of travel in days: <14; US census region of the Global TravEpiNet (GTEN) site: Northeast; type of GTEN site: academic center; Number of medical conditions: 0. Odds ratios, 95% confidence intervals, and *P* values obtained from multivariable logistic regression models to predict being vaccinated for pneumococcus. Abbreviations: CI, confidence interval; OR, odds ratio; US, United States; VFR, visiting friends or relatives.

We performed a multivariable logistic regression to further evaluate the factors that were associated with travelers not being vaccinated with the pneumococcal vaccine at the pretravel encounter (Figure 1). The analysis only included pneumococcal vaccine–eligible travelers as its base population. Compared to the reference groups for each variable, travelers were less likely to be vaccinated if they were seen at clinical sites outside the Northeast (Midwest: OR, 0.32 [95% CI, .25–.42]; South: OR, 0.53 [95% CI, .48–.57]; West: OR, 0.33 [95% CI, .30–.37]; *P* < .0001) or if they were seen at nonacademic centers (OR, 0.52 [95% CI, .46–.58]; *P* < .001). Additionally, travelers aged ≥65 years were more likely to be vaccinated (OR, 6.07 [95% CI, 5.65–6.51]; *P* < .001), as were travelers with at least 1 medical condition (OR, 1.61 [95% CI, 1.42–1.84]; *P* < .001).

### Clinical Approach to Influenza Vaccine–Eligible Travelers

Of the 116 865 patients receiving pretravel health consultations, 56 150 (48%) travelers had an indication for influenza vaccination (Table 2). Of these, 16 931 (30%) travelers declined vaccination for influenza, 15 658 (28%) were vaccinated during the visit, and 6955 (12%) were referred to their primary care physician (PCP) or other provider to receive a vaccine. An influenza vaccine was not offered to 16 606 (29%) travelers: for 8592 (15%) of these visits, the provider selected that an influenza vaccine was not indicated for the traveler's itinerary, while for 8014 (14%), the provider indicated that vaccine was not given because a vaccine was not available.

**Figure 2.**
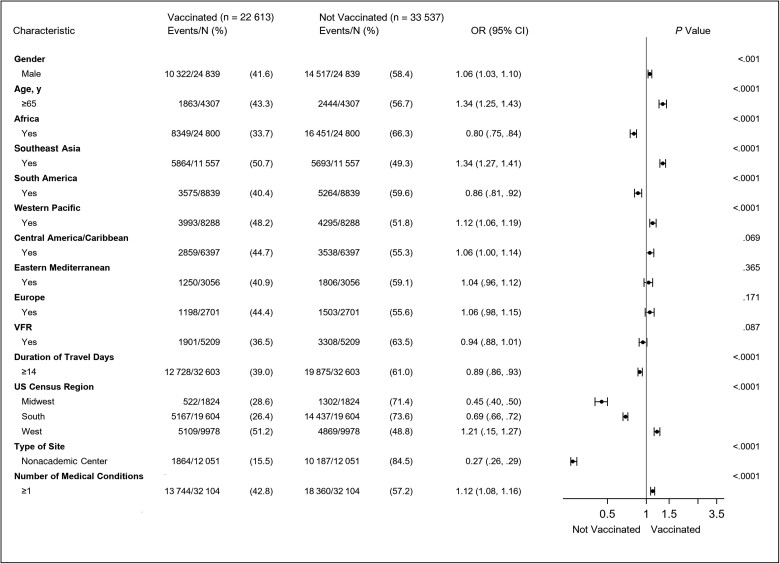
Forest plot depicting the results obtained from a multivariable logistic regression evaluating the relationship between traveler characteristics and receipt of influenza vaccine among persons eligible to receive the vaccine at the pretravel health encounter. Model included all characteristics shown in the figure. Reference categories: Gender: female; Age in years: <65; travel to each geographic region: no; Visiting friends and relatives: no; duration of travel in days: <14; US census region of the Global TravEpiNet (GTEN) site: Northeast; type of GTEN site: academic center; Number of medical conditions: 0. Odds ratios, 95% confidence intervals, and *P* values obtained from multivariable logistic regression models to predict being vaccinated for influenza. Abbreviations: CI, confidence interval; OR, odds ratio; US, United States; VFR, visiting friends or relatives.

We performed a multivariable logistic regression to further evaluate the traveler characteristics that were associated with not being vaccinated with an influenza vaccine at the pretravel encounter (Figure 2). Several factors were associated with lack of vaccination; the traveler being seen at a nonacademic center was the most highly associated factor.

### Travel to Regions With Active Influenza Transmission

**Figure 3.**
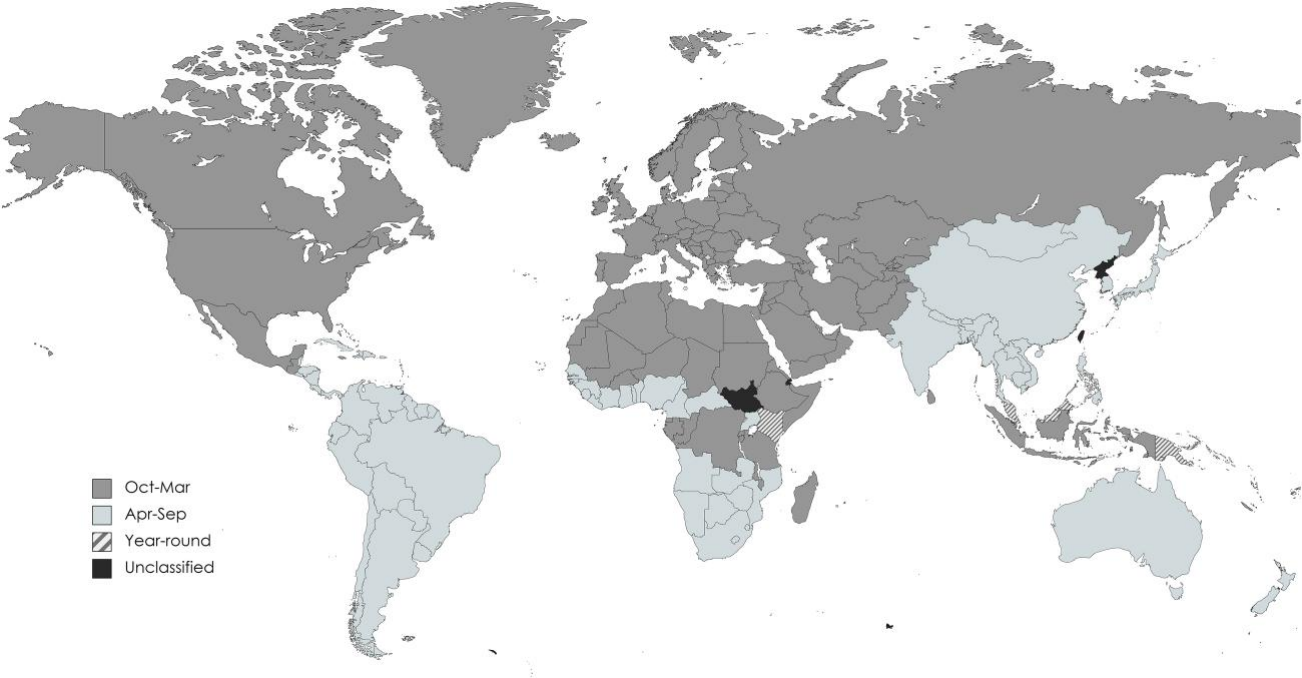
Global influenza seasonality as defined in this analysis (see Methods), based on epidimiological data published by the World Health Organization and other sources [13–17]. Map created using MapChart, used under a Creative Commons license (CC BY-SA 4.0).

To further evaluate the travelers who did not receive influenza vaccine at the pretravel health consultation, we evaluated influenza seasonality in the travel destination, as depicted in Figure 3 [17].

Among the 56 150 influenza vaccine–eligible travelers, 33 367 (59%) were traveling to a destination during its active influenza transmission period (Table 3; Figure 3). Among these, 7575 (23%) were vaccinated during the pretravel health consultation, 3962 (12%) were referred to another provider to receive an influenza vaccine, and 8767 (26%) declined vaccination. For 6251 (19%) travelers, the provider selected that a vaccine was not indicated for the traveler's itinerary as the reason for not administering a vaccine, while another 6610 (20%) travelers did not receive a vaccine because the provider stated that a vaccine was not available. Most of the travelers going to an area where influenza might be expected to be circulating who did not receive an influenza vaccine at the pretravel health encounter were traveling between the months of April–September (11 151/12 861 [87%]), a time period that overlaps with the June 30^th^ expiration date of seasonal influenza vaccine in the US.

**Table 3.** Travelers Eligible for Influenza Vaccination Who Were Traveling to a Destination During an Active Influenza Transmission Period in Global TravEpiNet, 2012–2022

Vaccination Status	Total	Active Transmission Time Period at Destination		
		Apr–Sep	Oct–Mar	Year-Round
Eligible travelers	33 367	23 388	5839	6103
Vaccinated or referred				
Administered during the visit	7575 (23)	4116 (18)	2549 (44)	1497 (25)
Referred to another provider for administration	3962 (12)	2548 (11)	840 (14)	823 (14)
Not vaccinated^a^				
Patient declined	8767 (26)	5433 (23)	2030 (35)	1798 (30)
Vaccine not available	6610 (20)	5938 (25)^b^	13 (<1)	1002 (16)
Not indicated for this itinerary	6251 (19)	5213 (22)^c^	373 (6)	947 (16)

Data are presented as No. (column %).

^a^Categories with <1000 travelers in all categories, which included “insufficient time to complete prior to departure” and “medical contraindication,” were also omitted.

^b^4162 (70%) travelers were seen in July or August.

^c^2000 (38%) travelers were seen in July or August.

## DISCUSSION

Addressing vaccine-preventable diseases is a key aspect of the pretravel health consultation. Our analysis demonstrates that travel medicine clinics contribute to the administration of vaccines against pneumococcus and influenza; 19% of vaccine-eligible travelers either received a pneumococcal vaccine or were referred to another provider to receive it, and 40% of travelers received influenza vaccination at their pretravel visit or were referred to another provider to receive it.

Importantly, there were missed opportunities for vaccination against pneumococcus and influenza at the pretravel health encounter. This was particularly remarkable in the case of pneumococcal vaccination, as approximately three-quarters of vaccine-eligible travelers did not receive the vaccine; the most common reason cited for not administering pneumococcal vaccination was that providers considered it not indicated for the traveler's itinerary. While the pneumococcal vaccine is not a travel-related vaccine per se, these travel clinic visits represent missed opportunities for vaccination, either during the visit or through referral to another provider.

There are several possible barriers to pneumococcal vaccination at the pretravel health encounter. One potential barrier is a lack of insurance coverage for administration of nontravel vaccines at a pretravel health encounter. It is also possible that some travel clinics may not stock pneumococcal vaccines or that travel clinic providers prioritize travel-related vaccines rather than vaccines unrelated to travel during the pretravel health encounter. Some travel medicine providers referred travelers to their PCP or another provider for pneumococcal vaccination; our analysis did not allow us to assess whether these recommendations were pursued. Increasing the administration of pneumococcal vaccine at the pretravel health encounter or with other non-PCP providers would be one strategy to improve vaccination rates for this routine vaccine [19].

In the case of the influenza vaccine, we conducted an additional analysis to assess missed opportunities for vaccination by including only patients traveling to a destination during the typical influenza season at that destination. Within this population, vaccine refusal (26%) was the most common explanation for the missed opportunity for vaccination. However, there were 2 additional barriers to influenza vaccination among this subgroup of travelers: lack of vaccine availability (20%) and the provider considering the vaccine as not indicated for the itinerary (19%). These 2 barriers were most common during travel visits that occurred between April and September. Notably, influenza vaccines expire June 30^th^ of each year and are generally not available in the US between July and late August [20]. Consequently, the lack of an available and appropriate influenza vaccine for Northern Hemisphere travelers is a barrier to vaccination for those traveling to areas with active influenza transmission in other regions of the globe, particularly those going to the Southern Hemisphere. While utilizing a Southern Hemisphere vaccine for cross-hemisphere travelers would be a reasonable strategy, these vaccines are not readily available in the US [21].

Our analysis also identified that the geographic location at which the pretravel visit took place had an impact on whether either vaccine was administered. Travelers seen at nonacademic centers or at centers in the South or the Midwest were less likely to receive either vaccine compared to travelers seen in academic centers or in centers in the Northeast. Reasons behind this observation are unclear. Patients aged ≥65 years were more likely to receive either vaccine; this may be related to either increased uptake of the vaccine from the traveler or increased recognition of the need for vaccination in this age group by the provider.

Our findings suggest that influenza vaccines are less likely to be offered to travelers during Northern Hemisphere summer months, even if the vaccines are available and influenza transmission is typically occurring at the traveler's destination. Improving practice in this area requires providers to recognize worldwide influenza seasonality. Generally, Southern Hemisphere travel during the Northern Hemisphere summer should prompt the provider to consider that the traveler may be entering an area with active influenza transmission. Furthermore, there are multiple destinations, most notably Kenya, Malaysia, and Singapore, among other tropical climates, in which influenza transmission is year-round [17]. Provider knowledge can be addressed by encouraging the use of available resources on the global epidemiology and seasonality of influenza, such as those provided by the World Health Organization or summarized elsewhere [13–17]. Including the destination-specific risk of influenza in resources frequently consulted by providers and travelers, such as the Centers for Disease Control and Prevention’s Travelers’ Health website [22], may be another way to raise awareness.

Our analysis suggests that making a Northern Hemisphere influenza vaccine available to travel clinics during the US summer months could be useful. One possible approach would be to permit travel clinics to utilize influenza vaccines beyond the June 30^th^ expiration date for eligible travelers whose itinerary involves an area with active transmission, until the new season's vaccines become available [17]; however, this would require regulatory changes, as providers cannot administer an expired vaccine. Another, somewhat more cumbersome solution would be to use Southern Hemisphere influenza vaccines as travel vaccines for cross-hemisphere travelers, which would also take into account the fact that travelers to the Southern Hemisphere may face influenza viruses not included in the Northern Hemisphere influenza vaccine [17, 23]. It should be noted that the 2 hemispheres' vaccines have often contained some of the same viruses. For instance, the 2023–2024 influenza vaccine formulation for the Northern Hemisphere differs from that for the 2023 Southern Hemisphere vaccine only in the H1N1 component [8]. In the current landscape of influenza vaccination, administering the vaccine approved at the traveler's point of origin remains the only available vaccination strategy to prevent influenza during travel.

Our study has limitations. Although GTEN is the largest multisite consortium of US travel medicine clinics, clinical practice in GTEN travel clinics may differ from other settings where pretravel health consultations are provided. Our study population may not be representative of all US travelers or of non-US-based international travelers, thereby limiting the generalizability of this study. Furthermore, pneumococcal vaccine eligibility for persons <65 years of age was based on broad categories of predisposing conditions, rather than specific diseases. Last, our evaluation did not include an analysis of coronavirus disease 2019 (COVID-19) vaccination, as these vaccines only became available during the last 18 months under analysis and data were limited due to a sharp decrease in travel medicine visits [24, 25]. Moreover, the overlap between the study period and the COVID-19 pandemic may have introduced variations in traveler behavior that could affect our findings.

In conclusion, travel medicine clinics contribute to the administration of routine vaccines against respiratory pathogens, in addition to travel-specific vaccines. Our multicenter observational study is the largest such study in departing US travelers, and we identified missed opportunities to provide travelers with vaccines recommended by ACIP for the prevention of respiratory illness. Improving administration and uptake of respiratory vaccines at the pretravel health encounter could be useful for public health.

## Notes

***Author contributions*.** Study conception and design: L. K., L. H. C., E. T. R., and R. C. L. Data collection: L. K. and S. R. R. Statistical analysis: S. R. R. Drafting the manuscript: L. K., L. H. C., and R. C. L. Manuscript review: All authors.

***Data availability*.** Data can be made available upon request from the authors.

***Financial support*.** This work was supported by the Centers for Disease Control and Prevention (grant number U01 CK000633).
